# The Predictive Value of Hemoglobin Glycation Index and Clonal Hematopoiesis of Indeterminate Potential Among AMI Patients—A Prospective Registry Study

**DOI:** 10.1111/1753-0407.70195

**Published:** 2026-02-16

**Authors:** Linghan Xue, Wenhao Dong, Jiannan Li, Runzhen Chen, Nan Li, Chen Liu, Peng Zhou, Yi Chen, Li Song, Xiaoxiao Zhao, Hongbing Yan, Hanjun Zhao

**Affiliations:** ^1^ Department of Cardiology Fuwai Hospital, National Center for Cardiovascular Diseases, Peking Union Medical College and Chinese Academy of Medical Sciences Beijing China; ^2^ Beijing Amcare Hospital Beijing China; ^3^ Coronary Heart Disease Center, Fuwai Hospital, Chinese Academy of Medical Sciences Beijing China

**Keywords:** acute coronary syndrome, clonal hematopoiesis of indeterminate potential, hemoglobin glycation index, mortality

## Abstract

**Aims:**

The objective of this research is to explore the combined effect of hemoglobin glycation index (HGI) and clonal hematopoiesis of indeterminate potential (CHIP) on all‐cause mortality among patients diagnosed with acute myocardial infarction (AMI).

**Methods:**

The presence of CHIP in peripheral blood cells was detected using deep targeted sequencing. AMI patients were divided into groups based on the median value of HGI and assessed for the effect of CHIP on all‐cause mortality using multivariable Cox regression and Kaplan–Meier analysis.

**Results:**

Multivariable Cox regression indicated that among patients with HGI above the median value, CHIP carriers exhibited a significantly higher all‐cause mortality (any CHIP, adjusted HR: 2.06 95% CI: 1.10–3.85; *p* = 0.023), with analysis of specific mutations identifying particularly high risks for TET2 (adjusted HR: 3.72, 95% CI: 1.37–10.09; *p* = 0.010) and TET2/ASXL1 co‐mutations (adjusted HR: 2.61, 95% CI: 1.08–6.30; *p* = 0.033). Kaplan–Meier analysis in the high‐HGI group confirmed that carriers of any CHIP (*p* < 0.001) and common CHIP (*p* = 0.019) had a higher risk of mortality than noncarriers.

**Conclusions:**

Our study reveals that HGI significantly modifies CHIP‐related all‐cause mortality risk, which provides a way for refined risk stratification in patients with AMI.

## Highlights

This study identifies a synergistic interaction between hemoglobin glycation index (HGI) and clonal hematopoiesis of indeterminate potential (CHIP). High HGI levels significantly amplify the mortality risk associated with CHIP in AMI patients, particularly for TET2 and ASXL1 mutations. This HGI‐CHIP composite indicator offers a novel insight for refined cardiovascular risk stratification.

## Graphical abstract

The hemoglobin glycation index (HGI) serves as a composite marker integrating both metabolic and genetic factors. Recent studies indicate that the interplay between HGI and clonal hematopoiesis of indeterminate potential (CHIP), mediated through chronic inflammation, is a key driver of coronary atherosclerosis and poor prognosis. This synergy underscores the importance of assessing integrated metabolic‐genetic risk profiles for better cardiovascular risk stratification and targeted intervention.

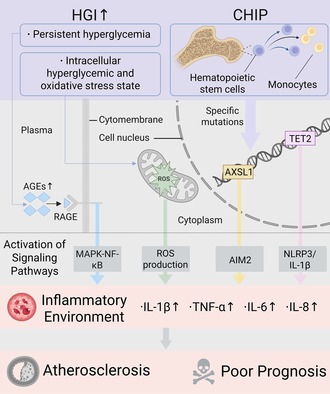

## 1 Introduction

Although the management of metabolic indicators is a crucial component in the prevention and treatment of Acute Coronary Syndrome (ACS) [1], advances in genome‐wide association studies (GWAS) are facilitating a shift toward a new paradigm guided by genetic information [2, 3]. Glycated hemoglobin (HbA1c) can reflect the average blood glucose level over the past 2 months and is essential to provide guidance on glucose management [4, 5]. Although HbA1c has been established as the gold standard for evaluating blood sugar control, mean blood glucose (MBG) levels account for approximately 60%–80% of the variance observed in hemoglobin A1c (HbA1c) levels [6]. This limitation arises because patients with similar blood glucose levels may have significantly different HbA1c levels, which is due to interindividual variations in passive hemoglobin glycation, glucose metabolism, and other factors [7, 8]. To quantify this individual variation, the hemoglobin glycation index (HGI) was introduced. HGI is defined as the difference between the measured and predicted HbA1c levels, where the predicted value is generated from a linear regression equation of observed MBG [9]. Importantly, several studies revealed that patients with abnormal HGI have a higher incidence of cardiovascular disease [10, 11, 12].

Clonal hematopoiesis of indeterminate potential (CHIP) can be characterized as a process initiated by somatic mutations leading to clonal expansion in the absence of cytopenia and dysplastic hematopoiesis [13]. Two critical studies have revealed that CHIP carriers are associated with an increased risk of coronary heart disease compared to noncarriers [14, 15]. This elevated risk is attributable primarily to the presence of specific mutations in genes including TET2, DNMT3A, and ASXL1. Moreover, the mechanism by which CHIP enhances coronary atherosclerosis is currently under investigation. For example, TET2‐deficient cells can activate the NLPR3 inflammasome and increase IL‐1β and IL‐6 expression, promoting atherosclerotic plaque destabilization [15].

While previous research on CHIP has focused primarily on general populations or patients with chronic coronary syndromes, studies focusing specifically on ACS populations remain scarce. Moreover, although CHIP carriers exhibit a higher incidence of major adverse cardiovascular events (MACEs) [16], the interaction between CHIP and HGI—a robust indicator for measuring an individual's blood glucose variability—has not been explored. A study found that HGI was associated with elevated inflammatory markers [17], suggesting that abnormal HGI and CHIP mutations may share overlapping mechanisms in driving increased inflammation. Furthermore, the insufficient control of traditional risk factors in previous studies further obscured the causal relationship between CHIP and outcomes. Therefore, we established a cohort to investigate the impact of CHIP on the prognosis in AMI patients with different HGI levels, aiming to examine the role of HGI in modifying the adverse outcomes linked to CHIP.

## 2 Methods

### 2.1 Study Design, Participants, and Sample Collection

**FIGURE 1:**
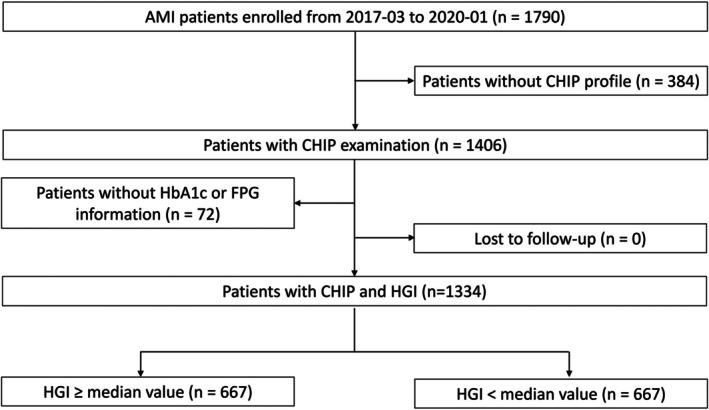
Study flow chart. AMI, acute myocardial infarction; CHIP, clonal hematopoiesis of indeterminate potential; FPG, fasting plasma glucose; HbA1c, hemoglobin A1c; HGI, hemoglobin glycation index.

Figure 1 outlines the design of the China, Risk, Genetics, Archiving, and Monograph (CRGAM) study, a prospective longitudinal study based on a specific population that enrolled adults aged 18 years or older with AMI who underwent primary percutaneous coronary intervention (PCI). The primary aim of this project is to construct cardiovascular risk prediction models based on traditional risk factors and to assess whether including genetic and biomarker data can enhance their prognostic accuracy. From March 2017 to January 2020, the study consecutively recruited eligible patients at Fuwai Hospital in Beijing, a national‐level cardiovascular disease center. These patients presented with AMI and required immediate coronary angiography. Further details regarding the study's objectives, participant eligibility, and cohort profile have been described in earlier publications [18].

**FIGURE 2:**
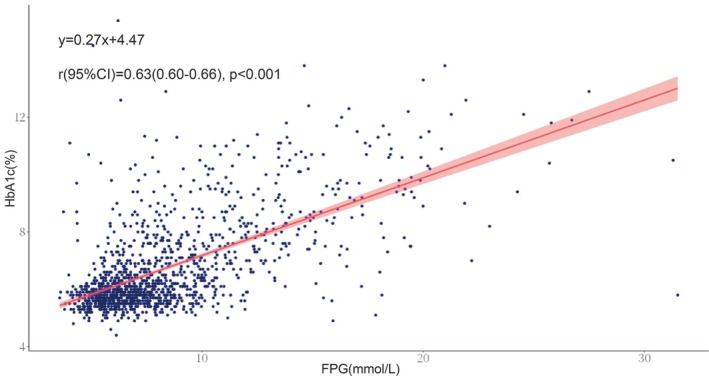
The linear regression of FPG on HbA1c. The red line indicates the fitted regression equation. FPG, fasting plasma glucose; HbA1c, hemoglobin A1c.

The diagnostic criteria of AMI were determined in accordance with international guidelines using clinical, electrocardiographic, biomarker, and imaging data. From March 2017 to January 2020, we consecutively enrolled patients with STEMI (ST‐elevation myocardial infarction) or NSTEMI (non‐ST‐elevation myocardial infarction) undergoing angiography. Treatment (conservative, PCI, or CABG) was decided by the heart team based on angiographic findings. Inclusion required: (1) Informed consent. (2) Completion of primary PCI (stent implantation with optional thrombus aspiration/balloon angioplasty). (3) Availability of complete data for CHIP and lipid parameters (triglyceride and LDL‐C). Exclusion criteria included: (1) Participant refusal; (2) Lost to follow‐up; (3) Unavailable CHIP data; (4) Comorbidities confounding the results (including inflammatory diseases and active or prior cancer); (5) Moderate to severe heart valve disease (except prior thoracoscopic ablation); (6) Severe chronic kidney disease (Stages 4 and 5, eGFR < 30 mL/min/1.73 m^2^); and (7) Moderate hepatic dysfunction. The study was ethically approved (Fuwai Hospital No. 2017‐866), followed the Declaration of Helsinki, and all participants provided informed consent. The HGI was derived from the difference between measured and predicted HbA1c levels [19], with the linear regression model predicting HbA1c from FPG: Predicted HbA1c = 0.27 × FPG (mmol/L) + 4.47 (*r* = 0.63, *p* < 0.001) (Figure 2).

### 2.2 Outcomes and Follow‐Up

The endpoint event was all‐cause death. Clinical follow‐up commenced immediately after hospital discharge, with an in‐person clinical examination scheduled at 1 month. For patients unable to attend, a validated questionnaire was administered via telephone by trained nurses. Subsequent follow‐ups were performed via telephone at 6 and 12 months, transitioning to annual assessments thereafter. All reported adverse events were adjudicated by an independent clinical event committee, and updated data were provided monthly to the research team.

### 2.3 Deep Targeted Sequencing of Clonal Hematopoiesis Mutations

Following informed consent, genomic DNA was extracted from arterial blood leukocytes (QIAamp DNA Blood Mini Kit). After quality control (gel electrophoresis, NanoDrop/Qubit), libraries were prepared using a custom 42‐gene CHIP panel (Agilent SureSelectXT) and subjected to enzymatic fragmentation, UMI ligation, and probe‐based enrichment. Qualified libraries (Q3 ≥ 80%) were sequenced on an Illumina NovaSeq 6000 (2 × 150 bp). Bioinformatics analysis involved UMI correction (Picard), variant calling (GATK), and annotation (ANNOVAR), with CHIP calls (VAF ≥ 2%) adhering to ICCS 2022 criteria. The median sequencing depth was 14 219×, with 97.2% of targets covered at ≥ 500× across 1406 samples (Method 1 in Table S1).

### 2.4 Statistical Analysis

Data are expressed as mean ± standard deviation for continuous variables and as number (percentage) for categorical variables. For assessing differences in clinical characteristics and CHIP prevalence between groups, the Student's *t*‐test (or the Mann–Whitney *U* test) and the chi‐square test (or Fisher's exact test) were used as appropriate. To evaluate the potential nonlinear relationship, the association of HGI with mortality was assessed via restricted cubic splines. Cox proportional hazards models were utilized to derive hazard ratios (HRs) with 95% confidence intervals (CIs) for clinical outcomes according to CHIP mutation status. The Kaplan–Meier curve was used to visually present all‐cause death across groups stratified by common CHIP mutations (VAF ≥ 2%), with between‐group differences compared using the log‐rank test. All statistical analyses were performed using R 4.4.1 (R Core Team, Vienna, Austria).

## 3 Results

### 3.1 Baseline Characteristics

Of the 1334 AMI patients undergoing primary PCI in the final cohort (Figure 1), CHIP mutations were identified in 147 participants (11.0%), and a subset of these (*n* = 83, 6.2%) carried a common CHIP mutation (VAF ≥ 2%). The predominant genes were DNMT3A (3.3%), TET2 (2.4%), and ASXL1 (1.3%), together representing 56.5% of all CHIP carriers. As demonstrated in Figure S1, HGI manifested a U‐shaped relationship with all‐cause mortality (cutoff: −0.229; overall *p* = 0.018, nonlinear *p* = 0.011). Relative to the low HGI group, high HGI patients demonstrated a distinct risk profile: a lower prevalence of diabetes, prior stroke, and MI, but a higher rate of previous PCI (all *p* < 0.05). They were also older and exhibited more adverse lipid and glycemic parameters, including lower HDL‐C, higher TG and HbA1c, and lower WBC (*p* < 0.001 for all) (Table S2).

Furthermore, Table 1 presents the demographic and clinical characteristics of patients with HGI below and above the median (*n* = 667 each), stratified by CHIP status in this prospective AMI cohort (*N* = 1334). Patients with any or common CHIP mutations were consistently older than noncarriers (all *p* < 0.05), in both low‐ and high‐HGI groups. In the low HGI cohort, CHIP carriers had a higher prevalence of prior stroke (any CHIP, *p* = 0.007) and a trend toward lower HDL‐C levels (any and common CHIP, *p* = 0.021 and *p* = 0.025, respectively). Conversely, in the high HGI cohort, carriers of CHIP exhibited significantly higher NT‐proBNP levels (common CHIP, *p* = 0.040), lower Killip classification (any and common CHIP, *p* = 0.002 and *p* < 0.001, respectively), and a greater likelihood of being prescribed clopidogrel at discharge (common CHIP, *p* = 0.021).

**TABLE 1:** Clinical features according to the presence of clonal hematopoiesis of indeterminate potential (variant allele fraction ≥ 2.0%) stratified by the median value of HGI.

Variables	HGI < median value (*n* = 667)			Common CHIP			HGI ≥ median value (*n* = 667)					
	Any CHIP						Any CHIP			Common CHIP		
	No (*n* = 595)	Yes (*n* = 72)	*p*1	No (*n* = 629)	Yes (*n* = 38)	*p*2	No (*n* = 592)	Yes (*n* = 75)	*p*3	No (*n* = 622)	Yes (*n* = 45)	*p*4
HGI	−0.67 (−1.01, −0.44)	−0.76 (−1.16, −0.45)	0.221	−0.67 (−1.02, −0.44)	−0.86 (−1.22, −0.57)	0.045	0.37 (0.04, 1.21)	0.43 (0.02, 1.12)	0.879	0.36 (0.04, 1.21)	0.44 (0.02, 1.03)	0.951
Patient characteristics												
Age (years)	59.00 (50.44, 67.00)	63.50 (54.75, 72.25)	0.006*	59.00 (50.48, 68.00)	64.50 (61.25, 73.32)	0.002*	61.00 (53.00, 68.00)	68.00 (61.50, 75.50)	< 0.001**	61.05 (53.00, 68.00)	68.00 (61.00, 73.00)	< 0.001**
Male, *n* (%)	493 (82.86)	56 (77.78)	0.286	518 (82.35)	31 (81.58)	0.903	478 (80.74)	57 (76.00)	0.331	501 (80.55)	34 (75.56)	0.417
BMI (kg/m^2^)	25.71 (23.36, 27.68)	25.33 (22.65, 27.83)	0.628	25.71 (23.37, 27.72)	24.74 (22.19, 27.50)	0.253	25.95 (23.53, 28.14)	25.26 (23.66, 27.27)	0.195	25.95 (23.53, 28.08)	25.25 (23.84, 27.44)	0.389
Past history												
Smoking, *n* (%)	440 (73.95)	46 (63.89)	0.070	462 (73.45)	24 (63.16)	0.166	422 (71.28)	52 (69.33)	0.726	442 (71.06)	32 (71.11)	0.994
Hypertension, *n* (%)	376 (63.19)	49 (68.06)	0.418	401 (63.75)	24 (63.16)	0.941	380 (64.19)	48 (64.00)	0.974	396 (63.67)	32 (71.11)	0.314
Dyslipidemia, *n* (%)	526 (88.40)	65 (90.28)	0.636	558 (88.71)	33 (86.84)	0.929	541 (91.39)	66 (88.00)	0.334	568 (91.32)	39 (86.67)	0.433
Diabetes mellitus, *n* (%)	99 (16.64)	14 (19.44)	0.549	105 (16.69)	8 (21.05)	0.487	298 (50.34)	37 (49.33)	0.870	307 (49.36)	28 (62.22)	0.096
Stroke, *n* (%)	66 (11.09)	16 (22.22)	0.007*	73 (11.61)	9 (23.68)	0.051	97 (16.41)	15 (20.00)	0.434	102 (16.43)	10 (22.22)	0.315
CKD, *n* (%)	42 (7.06)	6 (8.33)	0.693	44 (7.00)	4 (10.53)	0.621	39 (6.60)	5 (6.67)	1.000	41 (6.60)	3 (6.67)	1.000
MI, *n* (%)	84 (14.12)	14 (19.44)	0.228	90 (14.31)	8 (21.05)	0.254	115 (19.43)	15 (20.00)	0.906	120 (19.29)	10 (22.22)	0.632
PCI, *n* (%)	85 (14.29)	12 (16.67)	0.588	90 (14.31)	7 (18.42)	0.485	123 (20.78)	15 (20.00)	0.876	129 (20.74)	9 (20.00)	0.906
Laboratory data												
Total cholesterol (mmol/L)	4.21 (3.61, 5.05)	4.09 (3.55, 4.84)	0.321	4.20 (3.60, 5.05)	4.06 (3.71, 4.75)	0.245	4.20 (3.54, 4.92)	4.26 (3.58, 4.88)	0.869	4.20 (3.52, 4.89)	4.26 (3.67, 4.96)	0.447
LDL (mmol/L)	2.66 (2.06, 3.32)	2.55 (1.83, 3.14)	0.159	2.65 (2.04, 3.32)	2.62 (2.12, 3.00)	0.528	2.60 (2.00, 3.23)	2.58 (2.05, 3.32)	0.654	2.60 (2.00, 3.23)	2.59 (2.17, 3.36)	0.462
HDL (mmol/L)	1.08 (0.91, 1.28)	1.00 (0.89, 1.15)	0.021*	1.08 (0.91, 1.27)	0.97 (0.82, 1.15)	0.025*	1.00 (0.86, 1.17)	1.02 (0.89, 1.25)	0.444	1.00 (0.86, 1.17)	1.05 (0.91, 1.24)	0.225
TG (mmol/L)	1.35 (0.94, 1.88)	1.40 (0.96, 1.86)	0.725	1.36 (0.94, 1.90)	1.31 (0.90, 1.81)	0.666	1.52 (1.07, 2.18)	1.40 (0.94, 2.10)	0.202	1.51 (1.05, 2.17)	1.66 (1.15, 2.37)	0.680
Serum creatinine (μmol/L)	83.00 (71.53, 97.35)	82.75 (71.28, 92.81)	0.453	83.00 (71.50, 97.10)	83.25 (72.85, 91.61)	0.610	4.20 (3.54, 4.92)	4.26 (3.58, 4.88)	0.869	84.72 (72.04, 96.09)	81.80 (74.00, 96.60)	0.763
WBC (×10^9^/L)	9.61 (7.71, 12.11)	8.99 (7.37, 11.54)	0.128	9.55 (7.71, 12.06)	9.07 (7.39, 11.53)	0.286	8.73 (7.16, 10.49)	9.50 (7.39, 11.38)	0.212	8.77 (7.19, 10.59)	8.80 (7.36, 10.96)	0.683
hs‐CRP (mg/L)	5.43 (1.77, 10.66)	6.07 (2.50, 11.04)	0.423	5.48 (1.82, 10.70)	3.96 (1.83, 10.21)	0.814	5.67 (2.10, 10.88)	6.77 (2.28, 10.80)	0.671	5.64 (2.09, 10.84)	7.81 (2.69, 11.30)	0.199
FPG (mmol/L)	7.42 (6.12, 9.29)	7.78 (6.12, 9.67)	0.479	7.42 (6.12, 9.19)	8.52 (6.62, 11.05)	0.065	7.31 (5.69, 10.33)	7.06 (5.75, 9.79)	0.817	7.26 (5.71, 10.28)	7.30 (5.69, 9.99)	0.800
HbA1c (%)	5.70 (5.50, 6.10)	5.70 (5.40, 6.12)	0.845	5.70 (5.50, 6.10)	5.85 (5.50, 6.20)	0.428	7.10 (6.20, 8.60)	6.90 (6.20, 8.65)	0.831	7.10 (6.20, 8.60)	6.90 (6.30, 8.70)	0.889
cTnI (μg/L)	0.74 (0.09, 5.21)	1.52 (0.10, 4.62)	0.447	0.79 (0.09, 5.22)	1.21 (0.09, 3.52)	0.994	0.95 (0.11, 5.48)	0.93 (0.11, 3.77)	0.865	0.95 (0.11, 5.41)	0.93 (0.15, 3.80)	0.981
NT‐proBNP (pg/mL)	193.65 (59.97, 810.20)	280.85 (90.05, 773.23)	0.268	200.85 (60.35, 815.67)	231.65 (84.55, 619.98)	0.715	325.20 (77.20, 888.60)	428.70 (103.68, 1468.40)	0.070	325.20 (79.10, 905.90)	515.15 (117.10, 1406.02)	0.040*
LVEF (%)	55.00 (50.00, 60.00)	56.00 (49.50, 59.25)	0.873	55.00 (50.00, 60.00)	55.50 (48.00, 59.75)	0.839	55.00 (50.00, 59.25)	55.00 (48.00, 58.00)	0.281	55.00 (50.00, 59.00)	55.00 (48.00, 58.00)	0.288
HR (beats/min)	75.00 (65.00, 85.00)	74.50 (65.75, 86.00)	0.909	75.00 (65.50, 85.00)	69.50 (63.25, 81.25)	0.172	75.00 (64.00, 86.00)	75.00 (64.50, 86.00)	0.743	75.00 (64.00, 86.00)	75.00 (64.00, 93.00)	0.660
Systolic pressure (mmHg)	124 (109, 136)	125 (111, 138)	0.616	124 (109, 136)	126 (111, 137)	0.610	125 (112, 137)	130 (116, 141)	0.086	126 (112, 138)	127 (119, 138)	0.217
Diastolic pressure (mmHg)	78 (68, 88)	80 (70, 91)	0.268	78 (68, 89)	80 (70, 91)	0.450	78 (70, 87)	79 (73, 86)	0.454	78 (70, 87)	80 (76, 89)	0.101
Killip classification			0.325			0.141			0.002*			< 0.001**
1, *n* (%)	505 (85.02)	55 (76.39)		532 (84.71)	28 (73.68)		510 (88.85)	53 (76.81)		536 (88.89)	27 (67.50)	
2, *n* (%)	45 (7.58)	11 (15.28)		49 (7.80)	7 (18.42)		43 (7.49)	16 (23.19)		46 (7.63)	13 (32.50)	
3, *n* (%)	8 (1.35)	2 (2.78)		8 (1.27)	2 (5.26)		8 (1.39)	0 (0.00)		8 (1.33)	0 (0.00)	
4, *n* (%)	18 (3.03)	3 (4.17)		20 (3.18)	1 (2.63)		12 (2.09)	0 (0.00)		12 (1.99)	0 (0.00)	
Angiography data												
Culprit vessel						0.210			0.255			0.326
LAD, *n* (%)	260 (43.84)	26 (36.62)		273 (43.54)	13 (35.14)		245 (41.60)	25 (33.33)		252 (40.71)	18 (40.00)	
LCX, *n* (%)	70 (11.80)	10 (14.08)		75 (11.96)	5 (13.51)		100 (16.98)	12 (16.00)		105 (16.96)	7 (15.56)	
RCA, *n* (%)	225 (37.94)	30 (42.25)		241 (38.44)	14 (37.84)		198 (33.62)	33 (44.00)		214 (34.57)	17 (37.78)	
TIMI flow			0.993			0.869			0.914			0.857
0, *n* (%)	360 (65.45)	43 (68.25)		384 (65.98)	19 (61.29)		277 (52.46)	36 (53.73)		291 (52.43)	22 (55.00)	
1, *n* (%)	29 (5.27)	3 (4.76)		30 (5.15)	2 (6.45)		27 (5.11)	3 (4.48)		29 (5.23)	1 (2.50)	
2, *n* (%)	53 (9.64)	5 (7.94)		55 (9.45)	3 (9.68)		60 (11.36)	8 (11.94)		65 (11.71)	3 (7.50)	
3, *n* (%)	104 (18.91)	12 (19.05)		109 (18.73)	7 (22.58)		157 (29.73)	20 (29.85)		163 (29.37)	14 (35.00)	
Stent, *n* (%)	447 (81.57)	52 (82.54)	1.000	471 (81.21)	28 (90.32)	0.370	420 (79.70)	60 (89.55)	0.054	444 (80.14)	36 (90.00)	0.126
IABP, *n* (%)	36 (6.56)	6 (9.52)	0.485	38 (6.54)	4 (12.90)	0.199	23 (4.36)	1 (1.49)	0.427	23 (4.15)	1 (2.50)	0.923
Medical therapy												
ASA, *n* (%)	561 (94.44)	66 (91.67)	0.480	592 (94.27)	35 (92.11)	0.635	563 (95.10)	72 (96.00)	0.502	593 (95.34)	42 (93.33)	0.307
Clopidogrel, *n* (%)	287 (48.32)	41 (56.94)	0.349	305 (48.57)	23 (60.53)	0.315	307 (51.86)	47 (62.67)	0.094	323 (51.93)	31 (68.89)	0.021*
Ticagrelor, *n* (%)	292 (49.16)	31 (43.06)	0.607	307 (48.89)	16 (42.11)	0.547	278 (46.96)	27 (36.00)	0.122	291 (46.78)	14 (31.11)	0.063
ACEI/ARB/ARNI, *n* (%)	433 (72.90)	46 (63.89)	0.275	455 (72.45)	24 (63.16)	0.318	411 (69.43)	49 (65.33)	0.404	432 (69.45)	28 (62.22)	0.207
BB, *n* (%)	494 (83.16)	61 (84.72)	0.878	524 (83.44)	31 (81.58)	0.726	516 (87.16)	67 (89.33)	0.441	544 (87.46)	39 (86.67)	0.401
Statin, *n* (%)	568 (95.62)	66 (91.67)	0.211	598 (95.22)	36 (94.74)	0.768	560 (94.59)	72 (96.00)	0.427	588 (94.53)	44 (97.78)	0.136
Anticoagulant, *n* (%)	14 (2.36)	2 (2.86)	0.631	15 (2.40)	1 (2.63)	1.000	15 (2.53)	1 (1.33)	0.713	15 (2.41)	1 (2.22)	0.402

Abbreviations: ACEI, angiotensin‐converting enzyme inhibitor; ARB, angiotensin receptor blocker; ASA, acetylsalicylic acid (aspirin); BB, beta‐blocker; BMI, body mass index; CKD, chronic kidney disease; cTnI, cardiac troponin I; FPG, fasting plasma glucose; HbA1c, hemoglobin A1c; HDL, high‐density lipoprotein; HGI, hemoglobin glycation index; HR, heart rate; hs‐CRP, high‐sensitivity C‐reactive protein; IABP, intra‐aortic balloon pump; LDL, low‐density lipoprotein; LVEF, left ventricular ejection fraction; MI, myocardial infarction; PCI, percutaneous coronary intervention; TG, triglyceride; WBC, white blood cell.

* *p* < 0.05.

** *p* < 0.001.

The clinical characteristics of CHIP mutations varied by HGI status among diabetic patients. In the high HGI group, CHIP carriers had significantly higher mortality (*p* = 0.008) and worse clinical profiles, including older age (any and common CHIP, *p* < 0.001 and *p* = 0.026, respectively), higher blood pressure (SBP, any CHIP, *p* = 0.021; DBP, any CHIP, *p* = 0.046), and higher Killip classification (any and common CHIP, both *p* < 0.001). Conversely, in the low HGI group, CHIP exhibited no statistical difference with mortality, and the only significant difference was older age in common CHIP carriers (*p* = 0.038) (Table S3).

### 3.2 Comparison of HGI Levels According to CHIP Mutation Status: No Difference Between CHIP(+) And CHIP(−) Subgroups

**FIGURE 3:**
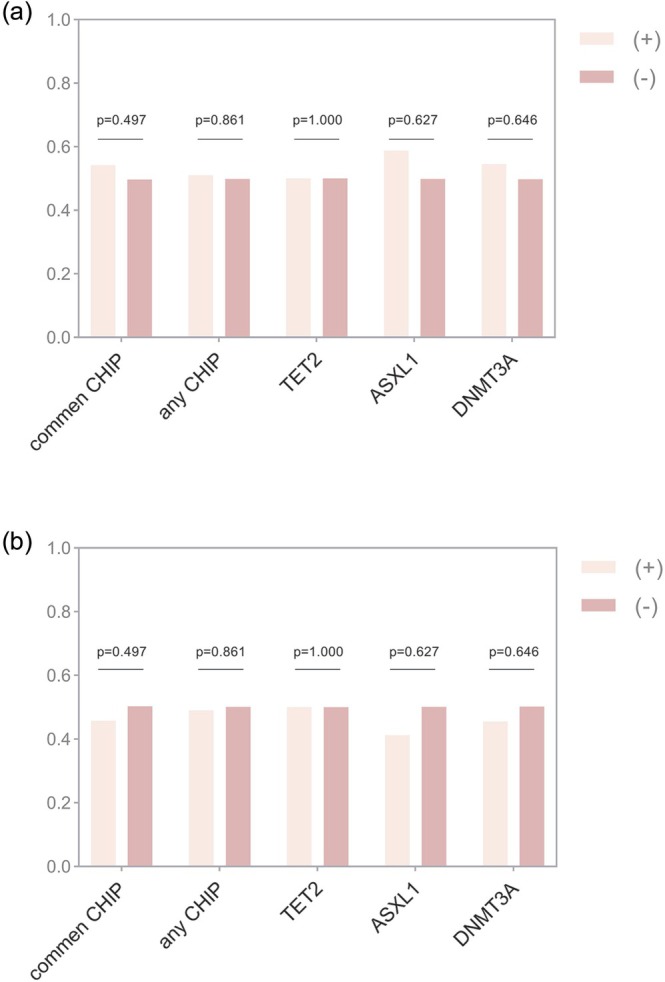
Proportions of patients with high or low HGI across CHIP subgroups. Bar graphs show the ratio of patients with high HGI (≥ median, a) and low HGI (< median, b) according to CHIP status, including any CHIP, common CHIP, and specific CHIP mutations (TET2, ASXL1, and DNMT3A). No statistically significant differences were observed between CHIP‐positive and CHIP‐negative groups in any subgroup (all *p* > 0.05). HGI, hemoglobin glycation index.

Figure 3 demonstrated that the ratio of patients with high HGI (≥ median) or low HGI (< median) across different CHIP‐positive and CHIP‐negative subgroups was comparable (all *p* > 0.05), indicating no statistical differences in the distribution of HGI categories between CHIP‐positive and CHIP‐negative individuals for any of the analyzed subgroups.

### 3.3 Elevated HGI in CHIP Is Associated With Increased Mortality Risk

Multivariable Cox regression analyses demonstrated that CHIP carriers with HGI levels above the median value had significantly higher all‐cause mortality in both the overall cohort and the DM cohort. As shown in Table 2, in the whole cohort, it was observed that among high HGI level (≥ median value) patients, CHIP carriers experienced significantly elevated all‐cause mortality (any CHIP, adjusted HR: 2.06, 95% CI: 1.10–3.85, *p* = 0.023). Analysis of specific mutations revealed that among patients with higher HGI, TET2 mutations exhibited the strongest association with mortality (adjusted HR: 3.72, 95% CI: 1.37–10.09, *p* = 0.010), and TET2/ASXL1 co‐mutations (adjusted HR: 2.61, 95% CI: 1.08–6.30, *p* = 0.033). However, no significant relationships between CHIP mutations and mortality were observed in patients with HGI below the median value. This association was consistent in the subgroup of patients with DM (Table 3). Among patients with DM and HGI ≥ median value, CHIP carriers had significantly elevated all‐cause mortality (any CHIP, HR: 2.72, 95% CI: 1.34–5.56, *p* = 0.006; common CHIP, HR: 2.31, 95% CI: 1.03–5.22, *p* = 0.043), with particularly elevated risks observed for TET2 mutations (HR: 4.78, 95% CI 1.87–12.19, *p* = 0.001) and TET2/ASXL1 co‐mutations (HR: 4.06, 95% CI: 1.71–9.65, *p* = 0.002). However, these associations became nonsignificant after additional adjustment for age, sex, and BMI.

**TABLE 2:** Multivariable Cox regression analyses of clonal hematopoiesis of indeterminate potential prevalence (variant allele fraction ≥ 2.0%) in HGI ≥ median value compared with HGI < median value in the entire study cohort.

HGI ≥ median value among all enrolled cohorts		HR (95% CI) of CHIP in HGI ≥ median value							
Variables	Death/CHIP (+)	Crude model	*p*1	Model 1	*p*2	Model 2	*p*3	Model 3	*p*4
Any CHIP mutations	18/75	2.63 (1.55–4.46)	**< 0.001** **	1.95 (1.06–3.60)	**0.033** *	1.95 (1.05–3.64)	**0.035** *	2.06 (1.10–3.85)	**0.023** *
Common CHIP mutations	10/45	2.18 (1.12–4.23)	**0.022** *	1.42 (0.65–3.09)	0.378	1.46 (0.67–3.22)	0.344	1.71 (0.78–3.76)	0.178
TET2/ASXL1	8/24	3.53 (1.70–7.36)	**< 0.001** **	2.67 (1.13–6.33)	**0.026** *	2.74 (1.16–6.47)	**0.022** *	2.61 (1.08–6.30)	**0.033** *
DNMT3A/TET2	9/40	2.19 (1.09–4.40)	**0.027** *	1.40 (0.61–3.23)	0.427	1.45 (0.62–3.37)	0.389	1.82 (0.78–4.24)	0.164
TET2	7/16	4.81 (2.21–10.47)	< 0.001**	3.66 (1.37–9.79)	**0.010** *	3.81 (1.43–10.19)	**0.008** *	3.72 (1.37–10.09)	**0.010** *
ASXL1	2/10	1.77 (0.43–7.20)	0.427	1.67 (0.37–7.53)	0.506	1.61 (0.35–7.33)	0.541	1.39 (0.29–6.63)	0.681
DNMT3A	2/24	0.70 (0.17–2.86)	0.622	0.43 (0.10–1.87)	0.259	0.42 (0.09–1.85)	0.250	0.62 (0.14–2.76)	0.534

HGI < median value among all enrolled cohorts		HR (95% CI) of CHIP in HGI < median value							
Variables	Death/CHIP (+)	Crude model	*p*1	Model 1	*p*2	Model 2	*p*3	Model 3	*p*4
Any CHIP mutations	9/72	1.62 (0.79–3.30)	0.184	1.11 (0.54–2.27)	0.772	1.11 (0.54–2.27)	0.776	1.34 (0.66–2.74)	0.422
Common CHIP mutations	4/38	1.26 (0.46–3.49)	0.654	0.61 (0.22–1.69)	0.343	0.55 (0.20–1.53)	0.254	0.60 (0.22–1.67)	0.332
TET2/ASXL1	4/23	2.19 (0.79–6.04)	0.132	1.31 (0.47–3.64)	0.599	1.31 (0.47–3.62)	0.606	1.46 (0.53–4.05)	0.465
DNMT3A/TET2	3/32	1.09 (0.34–3.48)	0.887	0.42 (0.13–1.34)	0.143	0.37 (0.11–1.17)	0.091	0.39 (0.12–1.26)	0.116
TET2	3/16	2.24 (0.70–7.18)	0.173	1.03 (0.32–3.31)	0.958	0.98 (0.31–3.14)	0.971	1.06 (0.33–3.41)	0.920
ASXL1	1/7	1.92 (0.26–13.85)	0.520	2.38 (0.33–17.26)	0.391	2.51 (0.35–18.21)	0.362	2.82 (0.39–20.48)	0.304
DNMT3A	0/20	—	—	—	—	—	—	—	—

*Note:* Model 1: adjusted for age, gender, hypertension, hyperlipidemia, and ejection fraction. Model 2: adjusted for Model 1 + history of MI, history of PCI, history of CABG. Model 3: adjusted for Model 2 + serum creatinine, Low‐density lipoprotein‐cholesterol. The bold values are represent statistically significant results with *P* < 0.05.

Abbreviations: CHIP, clonal hematopoiesis of indeterminate potential; CI, confidence interval; HGI, hemoglobin glycation index; HR, hazard ratio.

* *p* < 0.05.

** *p* < 0.001.

**TABLE 3:** Multivariable Cox regression analyses of clonal hematopoiesis of indeterminate potential prevalence (variant allele fraction ≥ 2.0%) in HGI ≥ median value compared with HGI < median value among the DM subgroup cohort.

HR (95% CI) of CHIP in HGI ≥ median value among DM cohort					
Variables	Death/CHIP (+)	Crude model	*p*1	Adjusted model	*p*2
Any CHIP mutations	10/37	2.72 (1.34–5.56)	**0.006** *	1.65 (0.78–3.49)	0.192
Common CHIP mutations	7/28	2.31 (1.03–5.22)	**0.043** *	1.49 (0.64–3.47)	0.355
TET2/ASXL1	6/15	4.06 (1.71–9.65)	**0.002** *	2.11 (0.83–5.37)	0.116
DNMT3A/TET2	6/24	2.30 (0.97–5.47)	0.059	1.49 (0.60–3.70)	0.396
TET2	5/11	4.78 (1.87–12.19)	**0.001** **	2.37 (0.83–6.81)	0.108
ASXL1	1/5	1.54 (0.21–11.20)	0.671	0.73 (0.10–5.43)	0.758
DNMT3A	1/13	0.58 (0.08–4.24)	0.594	0.56 (0.08–4.08)	0.568

HR (95% CI) of CHIP in HGI < median value among DM cohort					
Variables	Death/CHIP (+)	Crude model	*p*1	Adjusted model	*p*2
Any CHIP mutations	3/14	2.04 (0.57–7.33)	0.273	1.62 (0.41–6.49)	0.494
Common CHIP mutations	2/8	2.30 (0.51–10.30)	0.275	1.20 (0.21–6.89)	0.836
TET2/ASXL1	2/7	2.70 (0.60–12.10)	0.193	1.68 (0.31–9.03)	0.548
DNMT3A/TET2	1/5	1.72 (0.22–13.15)	0.602	0.72 (0.07–7.70)	0.788
TET2	1/4	2.23 (0.29–17.02)	0.441	1.25 (0.13–12.46)	0.849
ASXL1	1/3	3.04 (0.40–23.28)	0.284	2.03 (0.24–17.04)	0.516
DNMT3A	0/2	—	—	—	—

*Note:* Adjusted model includes sex, age, and BMI. The bold values are represent statistically significant results with *P* < 0.05.

Abbreviations: CHIP, clonal hematopoiesis of indeterminate potential; CI, confidence interval; DM, diabetic mellitus; HGI, hemoglobin glycation index; HR, hazard ratio.

* *p* < 0.05.

** *p* < 0.001.

We used multivariable Cox proportional hazards models to assess the relationship between HGI and all‐cause mortality in Table 4. In the overall patient cohort, no significant association was observed between HGI and mortality risk (adjusted HR: 1.08, 95% CI: 0.95–1.24, *p* = 0.248). Moreover, stratified analyses by CHIP status revealed that the association between HGI and increased mortality was significant in carriers of any CHIP (adjusted HR: 1.40, 95% CI: 1.07–1.84, *p* = 0.015) and particularly in carriers of common CHIP mutations (adjusted HR: 1.71, 95% CI: 1.21–2.42, *p* = 0.002). When analyzing specific driver genes, the effect was most prominent in patients with TET2 mutations (adjusted HR: 2.54, 95% CI: 1.33–4.85, *p* = 0.005, p for interaction = 0.026). We also observed a significant interaction for DNMT3A (*p* for interaction = 0.038), although the adjusted HR in the mutation carrier group was not statistically significant (adjusted HR: 2.06, 95% CI: 0.69–6.09; *p* = 0.193). No significant interaction was found for ASXL1 mutations.

**TABLE 4:** Multiple Cox proportional hazard models for the impact of HGI on the incidence of death.

Subgroup	*N* (%)	Crude HR (95% CI)	Crude *p*	Adjusted HR (95% CI)	Adjusted *p*	*p* for interaction^1^	*p* for interaction^2^
HGI was modeled as continuous (per 1‐unit increase)							
All patients	1334 (100.00)	1.08 (0.95–1.23)	0.214	1.08 (0.95–1.24)	0.248		
Any CHIP (−)	1187 (88.98)	1.02 (0.87–1.19)	0.836	1.01 (0.87–1.18)	0.875	0.169	0.066
Any CHIP (+)	147 (11.02)	1.22 (1.02–1.46)	**0.028** *	1.40 (1.07–1.84)	**0.015** *		
Commen CHIP (−)	1251 (93.78)	1.02 (0.88–1.18)	0.782	1.02 (0.88–1.18)	0.840	0.011	**0.010** *
Commen CHIP (+)	83 (6.22)	1.62 (1.18–2.21)	**0.003** *	1.71 (1.21–2.42)	**0.002** *		
TET2 (−)	1302 (97.60)	1.05 (0.91–1.20)	0.516	1.04 (0.90–1.20)	0.578	0.019*	0.026*
TET2 (+)	32 (2.40)	2.70 (1.42–5.13)	**0.002** *	2.54 (1.33–4.85)	**0.005** *		
DNMAT3A (−)	1290 (96.70)	1.06 (0.93–1.21)	0.370	1.06 (0.92–1.21)	0.426	**0.038** *	**0.038** *
DNMAT3A (+)	44 (3.30)	2.22 (1.01–4.89)	**0.048** *	2.06 (0.69–6.09)	0.193		
ASXL1 (−)	1317 (98.73)	1.08 (0.95–1.23)	0.234	1.08 (0.94–1.23)	0.268	0.743	0.726
ASXL1 (+)	17 (1.27)	1.26 (0.43–3.69)	0.680	1.45 (0.46–4.54)	0.525		

*Note:* HGI was modeled as continuous (per 1‐unit increase). The adjusted model includes age, sex, and BMI. The bold values are represent statistically significant results with *P* < 0.05. In 1= refers to the unadjusted interaction *p* value and 2= refers to the adjusted interaction *p* value.

Abbreviations: CHIP, clonal hematopoiesis of indeterminate potential; CI, confidence interval; HGI, hemoglobin glycation index; HR, hazard ratio.

* *p* < 0.05.

**FIGURE 4:**
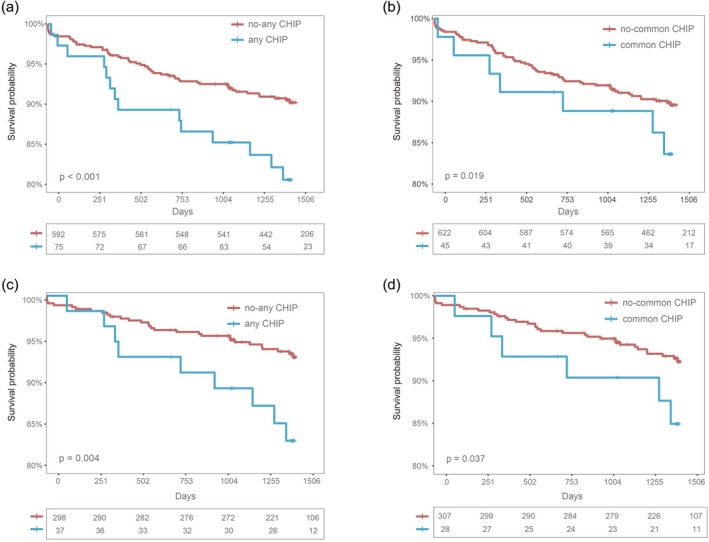
The Kaplan–Meier method was employed to estimate the mortality associated with the presence or absence of any CHIP and common CHIP, stratified by the median value of HGI in all patients and in patients with DM. Among individuals in the high HGI group, any CHIP (a) and common CHIP (b) mutation both experienced a higher risk of mortality, as well as in the DM patients (c and d), any and common CHIP mutation, respectively. CHIP, clonal hematopoiesis of indeterminate potential; DM, diabetes mellitus; HGI, hemoglobin glycation index.

Kaplan–Meier analyses corroborated these findings (Figure 4). In the overall cohort with high HGI (≥ median value), carriers of any CHIP (Figure 4a; *p* < 0.001) and common CHIP (Figure 4b; *p* = 0.019) mutations had significantly higher mortality compared to noncarriers. Similar patterns were observed in diabetic patients with high HGI, with any CHIP (Figure 4c; *p* = 0.004) and common CHIP (Figure 4d; *p* = 0.037) carriers also showing increased mortality. In contrast, no significant survival differences were observed between CHIP carriers and noncarriers in the low HGI (< median value) groups of either the overall cohort (Figure S2a, any CHIP, *p* = 0.180; Figure S2b, common CHIP, *p* = 0.653) or the DM cohort (Figure S2c, any CHIP, *p* = 0.262; Figure S2d, common CHIP, *p* = 0.261).

## 4 Discussion

In summary, this study reveals a synergistic effect between HGI and CHIP mutations in predicting the prognosis of patients with AMI. Our study shows that: (1) A high HGI level significantly increases the incidence of all‐cause mortality in CHIP carriers, predominantly driven by TET2/ASXL1, while no significant association was observed in the DM subgroup. (2) Elevated HGI level is associated with an increased mortality risk in CHIP carriers, whereas no such association was observed in CHIP‐negative individuals. (3) HGI exhibits a U‐shaped relationship with all‐cause death in AMI patients undergoing primary PCI. Our findings indicate that a composite indicator integrating HGI and CHIP, encompassing both metabolic dysfunction and somatic mutations, may offer a new approach to precisely identifying high‐risk AMI patients. This suggests that future risk management strategies should take such multidimensional risk factors into account.

By integrating both CHIP and HGI, our study revealed that among patients with an HGI ≥ median value, CHIP mutations were significantly associated with an increased risk of all‐cause mortality after adjusting for multiple confounding factors (Any CHIP mutation: adjusted HR = 2.0; TET2: adjusted HR = 3.72; TET2 or ASXL1: adjusted HR = 2.61). In contrast, this relationship was not observed in the low HGI group, which highlights the specificity of the synergistic effect between high HGI levels and CHIP mutations (predominantly TET2 and TET2/ASXL1). It is noteworthy that this conclusion is not applicable to the diabetic subgroup. Furthermore, a rising HGI level was associated with increased all‐cause mortality only in CHIP‐positive subgroups (any CHIP: adjusted HR = 2.06; TET2/ASXL1: adjusted HR = 2.61; TET2: adjusted HR = 3.72), with no statistically significant association observed in CHIP‐negative groups. We speculate that the lack of association between HGI and mortality in the CHIP‐negative subgroup may be explained by the absence of a preexisting inflammatory environment in these individuals. Our study is the first to identify a significant synergistic interaction between HGI and CHIP—a contrast to previous studies that examined these factors separately. For instance, Rhee et al. reported in their study of 2025 nondiabetic patients that elevated HGI conferred an increased risk of coronary artery calcification, independent of baseline HbA1c [11]. Similarly, in a cohort of 1248 patients, high HGI was identified as an independent risk factor for overall and cardiovascular diseases [20]. Concurrently, CHIP is increasingly recognized as an emerging predictor strongly linked to adverse prognosis in patients with coronary artery disease, an effect largely driven by mutations in genes such as TET2 [14]. A UK Biobank study involving 13 129 individuals with atherosclerotic cardiovascular disease established an independent association linking CHIP to adverse outcomes [16]. Our observations were consistent with the findings above. In addition, a U‐shaped relationship between HGI and all‐cause mortality was identified across the entire patient cohort, with higher values indicating chronic hyperglycemia or insulin resistance and lower values pointing to acute glycemic excursions or hypoglycemia‐related risks [20, 21, 22].

Our study investigated the synergistic association of specific CHIP mutations (e.g., TET2 and ASXL1) and abnormal HGI (particularly elevated levels) with mortality, providing novel insights into the underlying mechanisms. The following mechanisms may underlie this association. CHIP promotes a pro‐inflammatory state, thereby accelerating the development and progression of coronary atherosclerosis [15]. The loss‐of‐function (LOF) of TET2 drives the activation of the NLRP3 inflammasome/IL‐1β axis, which leads to the expression of a broad spectrum of pro‐inflammatory cytokines, including IL‐6 and IL‐8, ultimately leading to coronary atherosclerosis [15, 23, 24]. ASXL1 mutations are related to the pathogenesis of coronary atherosclerosis by upregulating the expression of the AIM2 inflammasome [25]. This process resulted in a chronic and low‐level inflammatory environment throughout the body and on the vascular walls. In addition, CHIP mutations have also been linked to glucose metabolic disorders. TET2‐deficient macrophages oversecrete IL‐1β, leading to insulin resistance via reducing expression of insulin receptor substrate 1 (IRS1) and glucose uptake [26, 27, 28].

Our study highlights that the HGI‐CHIP interaction may be linked through shared inflammatory pathways, metabolic dysregulation, and epigenetic alterations. As for HGI, a high level may point to an intrinsic defect in the patient's cells that impairs the ability to downregulate glucose uptake [29]. The resulting intracellular hyperglycemic and oxidative stress state leads to mitochondrial metabolic overload and excessive ROS production [30]. Persistent hyperglycemia drives the formation of advanced glycation end products (AGEs) [31]. These AGEs bind to receptor for AGEs (RAGE), subsequently activating the MAPK‐NF‐κB signaling pathway, which promotes the nuclear translocation of NF‐κB and the upregulated transcription of pro‐inflammatory cytokines (such as TNF‐α, IL‐6, and IL‐1β) [32, 33]. Concurrently, ROS derived from oxidative stress provides a secondary activation signal for the NLRP3 inflammasome, promoting IL‐1β maturation [34], which further reinforces NF‐κB activation and persistent inflammation. At the epigenetic level, high HGI reflects chronic hyperglycemia–induced “hyperglycemic memory” [35], which can sustain the activation of inflammation and oxidative stress‐related genes through persistent DNA methylation and histone modification changes. Chronic inflammation leads to endothelial dysfunction, which accelerates the progression of coronary atherosclerosis [36].

The effect of CHIP–HGI interaction on all‐cause mortality was not significant in the diabetic subgroup. In people with DM, key pro‐inflammatory pathways such as the NLRP3 inflammasome and NF‐κB may already be activated due to prolonged and high‐level stimulation [37, 38]. Furthermore, patients with diabetes typically receive more intensive management, for example, the widespread use of medication such as SGLT2 inhibitors and GLP‐1 receptor agonists [39, 40], which possess inherent anti‐inflammatory and cardiovascular protective properties that may counteract the additional risk imposed by CHIP and HGI.

## 5 Conclusions

In conclusion, by demonstrating that elevated HGI critically amplifies the prognostic impact of TET2/ASXL1 mutations on AMI mortality, this study offers new perspectives into the synergistic effect of CHIP mutations with HGI levels for refined risk stratification.

## Author Contributions

**Linghan Xue:** conceptualization, methodology, formal analysis, writing – original draft, funding acquisition. **Wenhao Dong:** investigation, data curation, writing – review and editing, visualization. **Jiannan Li:** validation, investigation, resources, data curation. **Runzhen Chen:** methodology, software, validation, data curation. **Nan Li:** investigation, resources, data curation. **Chen Liu:** investigation, resources, data curation. **Peng Zhou:** methodology, software, formal analysis. **Yi Chen:** validation, investigation, resources, project administration. **Li Song:** validation, supervision. **Xiaoxiao Zhao:** writing – review and editing, supervision, project administration, funding acquisition. **Hongbing Yan:** resources, writing – review and editing, supervision, project administration, funding acquisition. **Hanjun Zhao:** conceptualization, resources, writing – review and editing, supervision, project administration, funding acquisition.

## Funding

This study was supported by the Chinese Academy of Medical Sciences (CAMS) Innovation Fund for Medical Sciences (2023‐I2M‐C&T‐B‐069), the National Natural Science Foundation of China (82400410), and the Fundamental Research Fund for Central Universities (2025‐XHQN06).

## Ethics Statement

This study was ethically approved by the Ethics Committee of the Department of Cardiology, Fuwai Hospital, National Center for Cardiovascular Diseases, Peking Union Medical College (approval no. 2017‐866).

## Consent

All reported adverse events were adjudicated by an independent clinical event committee, and updated data were provided monthly to the research team.

## Conflicts of Interest

The authors declare no conflicts of interest.

## Supporting information


**Figure S1:** RCS curve of HGI and all‐cause death in all patients. CI, confidence interval; HGI, hemoglobin glycation index.
**Figure S2:** No similar associations between any (a) and common (b) CHIP mutation with all‐cause death were significantly noted in the setting of HGI less than the median level, as well as in the DM patients (c and d), any and common CHIP mutation, respectively.

**Table S1:** List of 42 clonal hematopoiesis‐associated genes analyzed by targeted sequencing.
**Table S2:** Clinical features according to the presence of clonal hematopoiesis of indeterminate potential (variant allele fraction ≥ 2.0%) stratified by the median value of HGI.
**Table S3:** Clinical features according to the presence of clonal hematopoiesis of indeterminate potential (variant allele fraction ≥ 2.0%) stratified by the median value of HGI among the DM cohort.

## Data Availability

Data sharing is not applicable to this article as no datasets were generated or analyzed during the current study.
