# Supplementary material for: The Predictive Value of Hemoglobin Glycation Index and Clonal Hematopoiesis of Indeterminate Potential Among AMI Patients—A Prospective Registry Study
Source: J Diabetes. 2026 Feb 16;18(2):e70195. doi: 10.1111/1753-0407.70195 (PMC12909099; doi:10.1111/1753-0407.70195)
Supplement: Supplementary file 1 — Figure S1: RCS curve of HGI and all‐cause death in all patients. CI, confidence interval; HGI, hemoglobin glycation index. Figure S2: No similar associations between any (a) and common (b) CHIP mutation with all‐cause death were significantly noted in the setting of HGI less than the median level, as well as in the DM patients (c and d), any and common CHIP mutation, respectively. [file JDB-18-e70195-s002.docx]

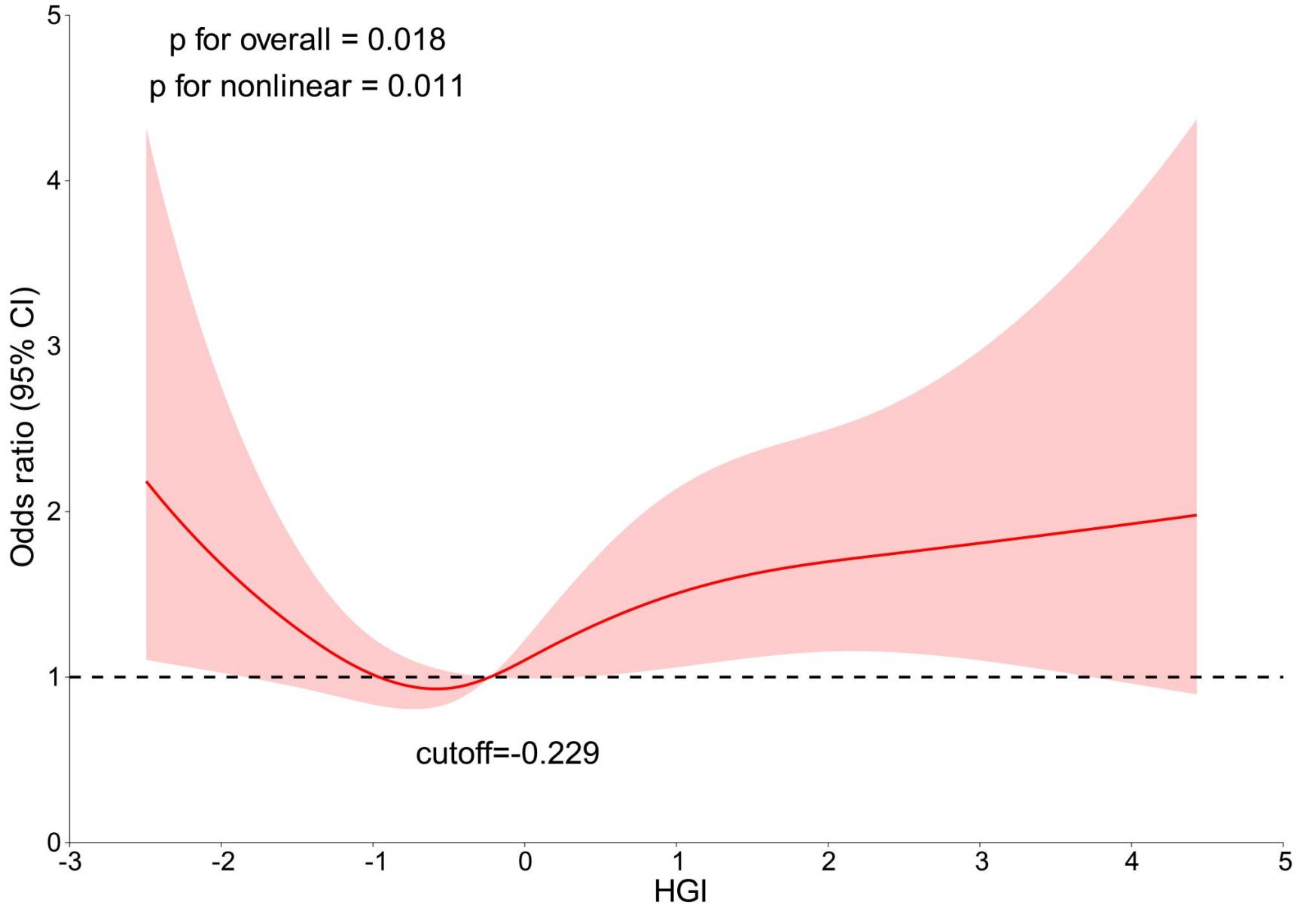


**Supplement Figure 1.** RCS curve of HGI and all-cause death in all patients. Abbreviations: CI, confidence interval; HGI, haemoglobin glycation index


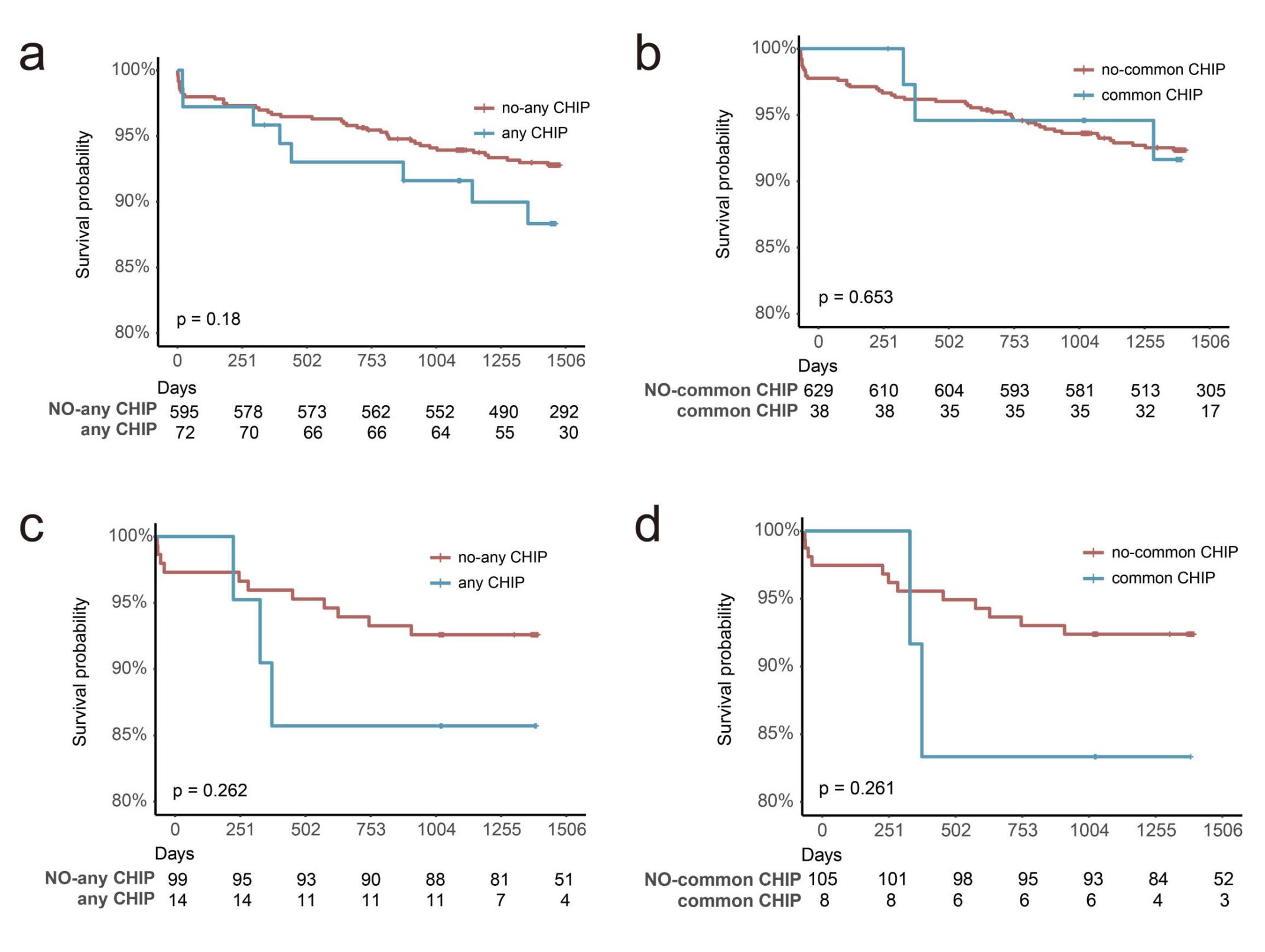


**Supplement Figure 2.** No similar associations between any (a) and common (b) CHIP mutation with all cause death were significantly noted in the setting of HGI less than median level, as well as in the DM patients (c and d, any and common CHIP mutation respectivel
