# Supplementary material for: The Predictive Value of Hemoglobin Glycation Index and Clonal Hematopoiesis of Indeterminate Potential Among AMI Patients—A Prospective Registry Study
Source: J Diabetes. 2026 Feb 16;18(2):e70195. doi: 10.1111/1753-0407.70195 (PMC12909099; doi:10.1111/1753-0407.70195)
Supplement: Supplementary file 2 — Table S1: List of 42 clonal hematopoiesis‐associated genes analyzed by targeted sequencing. Table S2: Clinical features according to the presence of clonal hematopoiesis of indeterminate potential (variant allele fraction ≥ 2.0%) stratified by the median value of HGI. Table S3: Clinical features according to the presence of clonal hematopoiesis of indeterminate potential (variant allele fraction ≥ 2.0%) stratified by the median value of HGI among the DM cohort. [file JDB-18-e70195-s001.docx]

**Supplement Method 1. Deep targeted sequencing of clonal hematopoiesis mutations**

Following informed consent, peripheral blood is drawn from the radial or femoral artery prior to heparinization and PCI. DNA is extracted from leukocytes, and its integrity and purity are verified by agarose gel electrophoresis (showing clear bands without trailing) and spectrophotometry (concentration≥20 ng/μL, total yield≥1μg, OD260/280 ratio of 1.8-2.0). A custom gene panel (Agilent, USA) for 42 CHIP-related genes is used[1]. The isolated DNA is fragmented, repaired, adenylated, and ligated with UMI adapters for library construction before being subjected to targeted sequencing by a commercial service (Tianhao, China). Bioinformatics analysis involves UMI correction, followed by the use of Picard for processing alignment files. ANNOVAR annotates all detected SNVs and InDels, cross-referencing them with population and disease databases to evaluate their frequency, function, and pathogenicity. CHIP mutations are identified by a VAF≥2.0%[2].

[1] Jaiswal S, Fontanillas P, Flannick J, et al. Agerelated clonal hematopoiesis associated with adverse outcomes. N Engl J Med. 2014;371:2488–2498.

[2] Jaiswal S, Libby P. Clonal haematopoiesis: connecting ageing and inflammation in cardiovascular disease. Nat Rev Cardiol 2020;17:137–44

**Supplement Table 1.** List of 42 clonal hematopoiesis-associated genes analyzed by targeted sequencing.

| *ASXL1* | *ASXL2* | *BCOR* | *BCORL1* | *BRCC3* | *CEBPA* | *CREBBP* | *CTCF* | *CUX1* | *DNMT3A* |
| --- | --- | --- | --- | --- | --- | --- | --- | --- | --- |
| *EP300* | *ETV6* | *EZH2* | *GATA1* | *GATA2* | *GATA3* | *GNB1* | *IDH2* | *IKZF2* | *IKZF3* |
| *JAK2* | *KDM6A* | *MPL* | *NF1* | *PDS5B* | *PHIP* | *PPM1D* | *PRPF40B* | *PTEN* | *RAD21* |
| *RUNX1* | *SETD2* | *SETDB1* | *SF1* | *SF3A1* | *SF3B1* | *SMC3* | *STAG1* | *STAG2* | *TET2* |
| *TP53* | *ZRSR2* |  |  |  |  |  |  |  |  |

**Supplement Table 2.** Clinical features according to the presence of clonal hematopoiesis of indeterminate potential (variant allele fraction ≥ 2.0%) stratified by the median value of HGI.

| Variables | Total | Group 1 | Group 2 | p value |
| --- | --- | --- | --- | --- |
|  | n = 1334 | HGI < median value  n = 667 | HGI ≥ median value  n = 667 |  |
| HGI | -0.23 (-0.68, 0.37) | -0.68 (-1.03, -0.44) | 0.37 (0.04, 1.20) | < 0.001** |
| Patient characteristics |  |  |  |  |
| Age (years) | 61.00 (52.00, 68.00) | 60.00 (51.00, 68.00) | 62.00 (53.60, 69.00) | 0.008* |
| Male, n (%) | 1084 (81.26) | 549 (82.31) | 535 (80.21) | 0.326 |
| BMI (kg/m^2^) | 25.71 (23.39, 27.78) | 25.66 (23.31, 27.70) | 25.83 (23.53, 28.07) | 0.073 |
| Past history |  |  |  |  |
| Smoking, n (%) | 374 (28.04) | 181 (27.14) | 193 (28.94) | 0.465 |
| Hypertension, n (%) | 481 (36.06) | 242 (36.28) | 239 (35.83) | 0.864 |
| Dyslipidemia, n (%) | 136 (10.19) | 76 (11.39) | 60 (9.00) | 0.148 |
| Diabetes mellitus, n (%) | 886 (66.42) | 554 (83.06) | 332 (49.78) | < 0.001** |
| Stroke, n (%) | 1139 (85.45) | 585 (87.71) | 554 (83.18) | 0.019* |
| CKD, n (%) | 1241 (93.10) | 619 (92.80) | 622 (93.39) | 0.671 |
| MI, n (%) | 1106 (82.91) | 569 (85.31) | 537 (80.51) | 0.020* |
| PCI, n (%) | 235 (17.62) | 97 (14.54) | 138 (20.69) | 0.003* |
| Laboratory data |  |  |  |  |
| Total cholesterol (mmol/L) | 4.20 (3.57, 4.95) | 4.19 (3.60, 5.04) | 4.20 (3.55, 4.91) | 0.452 |
| LDL (mmol/L) | 2.61 (2.01, 3.26) | 2.65 (2.04, 3.30) | 2.60 (2.00, 3.24) | 0.244 |
| HDL (mmol/L) | 1.04 (0.88, 1.23) | 1.07 (0.91, 1.27) | 1.01 (0.86, 1.18) | < 0.001** |
| TG (mmol/L) | 1.44 (1.01, 2.04) | 1.36 (0.94, 1.88) | 1.52 (1.06, 2.17) | < 0.001** |
| Serum creatinine (μmol/L) | 83.78 (71.89, 96.83) | 83.00 (71.51, 97.02) | 84.47 (72.22, 96.33) | 0.656 |
| WBC (×10⁹/L) | 9.15 (7.41, 11.37) | 9.52 (7.70, 11.98) | 8.78 (7.20, 10.65) | < 0.001** |
| hs-CRP (mg/L) | 5.62 (1.95, 10.77) | 5.47 (1.82, 10.70) | 5.82 (2.11, 10.87) | 0.257 |
| FPG (mmol/L) | 7.36 (5.91, 9.70) | 7.44 (6.12, 9.32) | 7.26 (5.70, 10.27) | 0.294 |
| HbA1c (%) | 6.10 (5.70, 7.40) | 5.70 (5.50, 6.10) | 7.10 (6.20, 8.60) | < 0.001** |
| cTnI (µg/L) | 0.90 (0.10, 5.17) | 0.81 (0.09, 5.17) | 0.95 (0.11, 5.13) | 0.318 |
| NT-proBNP (pg/mL) | 263.50 (69.30, 876.95) | 204.35 (61.30, 801.10) | 335.20 (82.20, 922.70) | 0.005 |
| LVEF (%) | 55.00 (50.00, 59.00) | 55.00 (50.00, 60.00) | 55.00 (49.00, 59.00) | 0.616 |
| HR (beats/min) | 75.00 (65.00, 85.00) | 75.00 (65.00, 85.00) | 75.00 (64.00, 86.00) | 0.994 |
| Systolic pressure (mmHg) | 125.00 (111.00, 137.00) | 124.00 (109.00, 136.00) | 126.00 (113.00, 138.00) | 0.099 |
| Diastolic pressure (mmHg) | 78.00 (70.00, 88.00) | 78.00 (68.00, 89.00) | 78.00 (70.00, 87.00) | 0.765 |
| Killip classification |  |  |  | 0.799 |
| 1, n (%) | 1123 (84.37) | 560 (84.08) | 563 (84.66) |  |
| 2, n (%) | 115 (8.64) | 56 (8.41) | 59 (8.87) |  |
| 3, n (%) | 18 (1.35) | 10 (1.50) | 8 (1.20) |  |
| 4, n (%) | 33 (2.48) | 21 (3.15) | 12 (1.80) |  |
| Angiography data |  |  |  |  |
| Culprit vessel |  |  |  | 0.077 |
| LAD, n (%) | 3 (0.23) | 1 (0.15) | 2 (0.30) |  |
| LCX, n (%) | 192 (14.46) | 80 (12.05) | 112 (16.87) |  |
| RCA, n (%) | 486 (36.60) | 255 (38.40) | 231 (34.79) |  |
| TIMI flow |  |  |  | < 0.001** |
| 0, n (%) | 716 (59.27) | 403 (65.74) | 313 (52.61) |  |
| 1, n (%) | 62 (5.13) | 32 (5.22) | 30 (5.04) |  |
| 2, n (%) | 126 (10.43) | 58 (9.46) | 68 (11.43) |  |
| 3, n (%) | 293 (24.25) | 116 (18.92) | 177 (29.75) |  |
| stent, n (%) | 979 (81.24) | 499 (81.67) | 480 (80.81) | 0.712 |
| IABP, n (%) | 66 (5.47) | 42 (6.86) | 24 (4.04) | 0.037* |
| Medical therapy |  |  |  |  |
| ASA, n (%) | 1262 (94.67) | 627 (94.14) | 635 (95.20) | 0.559 |
| Clopidogrel, n (%) | 682 (51.16) | 328 (49.25) | 354 (53.07) | 0.341 |
| Ticagrelor, n (%) | 628 (47.11) | 323 (48.50) | 305 (45.73) | 0.476 |
| ACEI/ARB/ARNI, n (%) | 939 (70.44) | 479 (71.92) | 460 (68.97) | 0.371 |
| BB, n (%) | 1138 (85.37) | 555 (83.33) | 583 (87.41) | 0.109 |
| Statin, n (%) | 1266 (94.97) | 634 (95.20) | 632 (94.75) | 0.674 |
| Anticoagulant, n (%) | 32 (2.40) | 16 (2.41) | 16 (2.40) | 0.713 |

Abbreviations: HGI, Hemoglobin Glycation Index; BMI, body mass index; CKD, chronic kidney disease; MI, myocardial infarction; PCI, percutaneous coronary intervention; LDL, low-density lipoprotein; HDL, high-density lipoprotein; TG, triglyceride; WBC, white blood cell; hs-CRP, high-sensitivity C-reactive protein; FPG, fasting plasma glucose; HbA1c, haemoglobin A1c; cTnI, cardiac troponin I; LVEF, left ventricular ejection fraction; HR, heart rate; IABP, intra-aortic balloon pump; ASA, acetylsalicylic acid (aspirin); ACEI, angiotensin-converting enzyme inhibitor; ARB, angiotensin receptor blocker; BB, beta-blocker

*p < 0.05, **p < 0.001

**Supplement Table 3.** Clinical features according to the presence of clonal hematopoiesis of indeterminate potential (variant allele fraction≥2.0%) stratified by the median value of HGI among DM cohort

| **Variables** | **DM patients with HGI < median level (*n* = 113)** | | |  | | | **DM patients with HGI ≥ median level (*n* = 335)** | | |  | | |
| --- | --- | --- | --- | --- | --- | --- | --- | --- | --- | --- | --- | --- |
|  | **Any CHIP** | | | **Common CHIP** | | | **Any CHIP** | | | **Common CHIP** | | |
|  | **No (n = 99)** | **Yes (n = 14)** | **p1** | **No (n = 105)** | **Yes (n = 8)** | **p2** | **No (n = 298)** | **Yes (n = 37)** | **p3** | **No (n = 307)** | **Yes (n = 28)** | **p4** |
| Death, n (%) | 11 (11.11) | 3 (21.43) | 0.507 | 12 (11.43) | 2 (25.00) | 0.258 | 31 (10.40) | 10 (27.03) | 0.008* | 34 (11.07) | 7 (25.00) | 0.064 |
| HGI | -0.76 (-1.10, -0.48) | -0.98 (-1.07, -0.71) | 0.122 | -0.76 (-1.08, -0.49) | -1.04 (-1.34, -0.88) | 0.096 | 1.01 (0.35, 1.96) | 0.92 (0.44, 1.90) | 0.794 | 1.02 (0.35, 1.96) | 0.90 (0.44, 1.57) | 0.466 |
| Patient characteristics |  |  |  |  |  |  |  |  |  |  |  |  |
| Age (years) | 62.00 (55.50, 68.00) | 62.50 (55.75, 78.25) | 0.333 | 62.00 (55.00, 68.00) | 75.00 (62.00, 81.25) | 0.038* | 62.00 (54.00, 68.00) | 69.00 (62.90, 78.00) | < 0.001** | 63.00 (54.00, 68.00) | 67.59 (60.50, 73.50) | 0.026* |
| Male, n (%) | 76 (76.77) | 10 (71.43) | 0.917 | 80 (76.19) | 6 (75.00) | 1.000 | 233 (78.19) | 25 (67.57) | 0.148 | 80 (76.19) | 6 (75.00) | 1.000 |
| BMI (kg/m^2^) | 25.66 (23.55, 27.77) | 25.33 (22.11, 26.15) | 0.329 | 25.66 (23.60, 27.78) | 23.27 (21.21, 25.42) | 0.060 | 25.71 (23.44, 27.78) | 25.71 (23.44, 27.46) | 0.789 | 25.71 (23.38, 27.78) | 25.83 (23.49, 27.61) | 0.963 |
| Past history |  |  |  |  |  |  |  |  |  |  |  |  |
| Smoking, n (%) | 72 (72.73) | 6 (42.86) | 0.051 | 74 (70.48) | 4 (50.00) | 0.417 | 208 (69.80) | 22 (59.46) | 0.201 | 213 (69.38) | 17 (60.71) | 0.344 |
| Hypertension, n (%) | 74 (74.75) | 9 (64.29) | 0.613 | 78 (74.29) | 5 (62.50) | 0.755 | 203 (68.12) | 29 (78.38) | 0.202 | 210 (68.40) | 22 (78.57) | 0.264 |
| Dyslipidemia, n (%) | 95 (95.96) | 12 (85.71) | 0.160 | 100 (95.24) | 7 (87.50) | 0.363 | 276 (92.62) | 36 (97.30) | 0.473 | 285 (92.83) | 27 (96.43) | 0.742 |
| Stroke, n (%) | 18 (18.18) | 4 (28.57) | 0.577 | 20 (19.05) | 2 (25.00) | 1.000 | 69 (23.15) | 8 (21.62) | 0.834 | 72 (23.45) | 5 (17.86) | 0.501 |
| CKD, n (%) | 12 (12.12) | 2 (14.29) | 1.000 | 13 (12.38) | 1 (12.50) | 1.000 | 22 (7.38) | 2 (5.41) | 0.919 | 22 (7.17) | 2 (7.14) | 1.000 |
| MI, n (%) | 14 (14.14) | 5 (35.71) | 0.101 | 17 (16.19) | 2 (25.00) | 0.879 | 64 (21.48) | 9 (24.32) | 0.692 | 66 (21.50) | 7 (25.00) | 0.667 |
| PCI, n (%) | 14 (14.14) | 4 (28.57) | 0.322 | 16 (15.24) | 2 (25.00) | 0.821 | 69 (23.15) | 7 (18.92) | 0.562 | 71 (23.13) | 5 (17.86) | 0.524 |
| Laboratory data |  |  |  |  |  |  |  |  |  |  |  |  |
| Total cholesterol (mmol/L) | 4.09 (3.46, 4.87) | 3.91 (3.16, 4.08) | 0.137 | 4.04 (3.41, 4.66) | 3.98 (3.23, 4.14) | 0.481 | 4.19 (3.53, 4.92) | 3.97 (3.56, 4.82) | 0.494 | 4.19 (3.53, 4.88) | 4.01 (3.58, 4.86) | 0.771 |
| LDL (mmol/L) | 2.46 (1.88, 3.08) | 2.24 (1.69, 2.60) | 0.229 | 2.45 (1.83, 3.02) | 2.50 (1.99, 2.69) | 0.845 | 2.56 (1.99, 3.24) | 2.44 (2.04, 3.26) | 0.686 | 2.55 (2.00, 3.24) | 2.42 (2.03, 3.28) | 0.807 |
| HDL (mmol/L) | 1.05 (0.89, 1.27) | 0.99 (0.78, 1.19) | 0.296 | 1.04 (0.89, 1.26) | 0.95 (0.70, 1.21) | 0.235 | 1.00 (0.85, 1.15) | 1.02 (0.85, 1.13) | 0.726 | 0.99 (0.84, 1.14) | 1.06 (0.91, 1.16) | 0.240 |
| TG (mmol/L) | 1.38 (1.09, 1.93) | 1.48 (1.25, 1.68) | 0.676 | 1.39 (1.10, 1.91) | 1.40 (1.21, 1.71) | 0.832 | 1.58 (1.08, 2.27) | 1.43 (1.15, 2.04) | 0.723 | 1.54 (1.08, 2.26) | 1.61 (1.15, 2.19) | 0.950 |
| Serum creatinine (μmol/L) | 83.49 (73.72, 97.41) | 84.40 (79.23, 90.02) | 0.828 | 83.49 (73.74, 96.70) | 84.40 (78.42, 87.98) | 0.805 | 84.38 (71.89, 97.80) | 83.10 (73.19, 98.00) | 0.743 | 84.47 (71.90, 98.00) | 82.45 (74.53, 96.95) | 0.747 |
| WBC (×10⁹/L) | 9.94 (8.24, 12.79) | 9.71 (8.97, 13.30) | 0.682 | 9.94 (8.21, 12.83) | 9.43 (9.22, 12.65) | 0.724 | 8.80 (7.21, 10.97) | 8.87 (6.94, 10.70) | 0.951 | 8.80 (7.20, 10.93) | 8.94 (7.08, 11.01) | 0.848 |
| hs-CRP (mg/L) | 6.48 (2.40, 11.52) | 9.56 (3.38, 11.88) | 0.356 | 6.48 (2.53, 11.51) | 10.22 (5.39, 12.17) | 0.293 | 5.91 (2.29, 11.22) | 9.08 (3.14, 11.04) | 0.268 | 5.98 (2.25, 11.20) | 8.46 (3.44, 11.18) | 0.270 |
| FPG (mmol/L) | 11.27 (9.25, 15.12) | 12.25 (8.94, 13.75) | 0.831 | 11.13 (9.11, 15.11) | 13.07 (11.52, 13.44) | 0.658 | 9.57 (7.55, 12.91) | 9.03 (7.08, 13.72) | 0.672 | 9.57 (7.53, 12.91) | 9.19 (7.10, 13.99) | 0.814 |
| HbA1c (%) | 6.70 (6.15, 7.50) | 6.40 (6.03, 7.15) | 0.342 | 6.70 (6.10, 7.50) | 6.70 (6.13, 7.15) | 0.583 | 8.30 (7.50, 9.70) | 8.30 (7.20, 9.50) | 0.540 | 8.30 (7.50, 9.70) | 8.10 (6.90, 9.50) | 0.376 |
| CTnI (µg/L) | 0.85 (0.11, 6.27) | 0.68 (0.09, 3.04) | 0.682 | 0.85 (0.10, 6.10) | 0.52 (0.09, 1.35) | 0.568 | 1.16 (0.14, 7.75) | 0.81 (0.17, 1.59) | 0.268 | 1.11 (0.15, 7.48) | 0.82 (0.23, 1.65) | 0.410 |
| NT-proBNP (pg/mL) | 331.40 (80.65, 1349.20) | 359.75 (128.27, 1759.62) | 0.641 | 331.40 (81.80, 1274.60) | 495.45 (121.07, 2448.88) | 0.650 | 394.00 (83.60, 1096.10) | 439.70 (120.10, 1376.30) | 0.275 | 395.20 (89.10, 1102.03) | 656.15 (106.62, 1135.85) | 0.297 |
| LVEF (%) | 55.00 (48.50, 58.00) | 50.50 (45.75, 58.50) | 0.685 | 54.00 (48.00, 58.00) | 56.00 (49.50, 60.00) | 0.452 | 55.00 (48.00, 58.00) | 55.00 (48.00, 59.00) | 0.792 | 55.00 (48.00, 58.00) | 53.50 (47.50, 58.25) | 0.561 |
| HR (beats/min) | 80.00 (69.50, 90.50) | 71.00 (66.00, 82.50) | 0.135 | 78.00 (69.00, 90.00) | 72.50 (66.00, 77.50) | 0.258 | 76.00 (66.00, 87.00) | 75.00 (67.00, 92.00) | 0.540 | 76.00 (66.00, 87.00) | 75.00 (67.00, 93.75) | 0.703 |
| Systolic pressure (mmHg) | 127.00 (110.50, 140.50) | 133.00 (112.00, 134.75) | 0.797 | 127.00 (112.00, 140.00) | 133.50 (109.25, 139.25) | 0.818 | 125.50 (113.00, 138.00) | 131.00 (125.00, 141.00) | 0.021* | 126.00 (114.00, 139.00) | 128.50 (119.00, 137.25) | 0.362 |
| Diastolic pressure (mmHg) | 80.00 (69.00, 88.00) | 78.50 (67.75, 89.50) | 0.810 | 80.00 (68.00, 88.00) | 78.50 (70.75, 87.75) | 0.991 | 76.50 (70.00, 86.25) | 80.00 (76.00, 89.00) | 0.046* | 77.00 (70.00, 87.00) | 80.50 (76.00, 87.50) | 0.107 |
| Killip classification |  |  | 0.273 |  |  | 0.124 |  |  | < 0.001** |  |  | < 0.001** |
| 1, n (%) | 80 (80.81) | 9 (64.29) |  | 85 (80.95) | 4 (50.00) |  | 251 (84.80) | 21 (56.76) |  | 258 (84.59) | 14 (50.00) |  |
| 2, n (%) | 11 (11.11) | 4 (28.57) |  | 12 (11.43) | 3 (37.50) |  | 27 (9.12) | 11 (29.73) |  | 28 (9.18) | 10 (35.71) |  |
| 3, n (%) | 3 (3.03) | 1 (7.14) |  | 3 (2.86) | 1 (12.50) |  | 6 (2.03) | 0 (0.00) |  | 6 (1.97) | 0 (0.00) |  |
| 4, n (%) | 4 (4.04) | 0 (0.00) |  | 4 (3.81) | 0 (0.00) |  | 4 (1.35) | 0 (0.00) |  | 4 (1.31) | 0 (0.00) |  |
| Angiography data |  |  |  |  |  |  |  |  |  |  |  |  |
| Culprit vessel |  |  | 0.013 |  |  | 0.004 |  |  | 0.438 |  |  | 0.636 |
| LAD, n (%) | 47 (47.96) | 3 (21.43) |  | 49 (47.12) | 1 (12.50) |  | 130 (44.07) | 14 (37.84) |  | 132 (43.42) | 12 (42.86) |  |
| LCX, n (%) | 7 (7.14) | 5 (35.71) |  | 8 (7.69) | 4 (50.00) |  | 40 (13.56) | 6 (16.22) |  | 42 (13.82) | 4 (14.29) |  |
| RCA, n (%) | 40 (40.82) | 5 (35.71) |  | 43 (41.35) | 2 (25.00) |  | 103 (34.92) | 14 (37.84) |  | 107 (35.20) | 10 (35.71) |  |
| TIMI flow |  |  | 0.909 |  |  | 0.849 |  |  | 0.653 |  |  | 0.675 |
| 0, n (%) | 64 (68.09) | 9 (81.82) |  | 68 (68.69) | 5 (83.33) |  | 134 (50.76) | 16 (48.48) |  | 136 (50.00) | 14 (56.00) |  |
| 1, n (%) | 2 (2.13) | 0 (0.00) |  | 2 (2.02) | 0 (0.00) |  | 17 (6.44) | 2 (6.06) |  | 19 (6.99) | 0 (0.00) |  |
| 2, n (%) | 13 (13.83) | 1 (9.09) |  | 14 (14.14) | 0 (0.00) |  | 33 (12.50) | 2 (6.06) |  | 33 (12.13) | 2 (8.00) |  |
| 3, n (%) | 15 (15.96) | 1 (9.09) |  | 15 (15.15) | 1 (16.67) |  | 74 (28.03) | 13 (39.39) |  | 78 (28.68) | 9 (36.00) |  |
| stent, n (%) | 14 (14.14) | 4 (28.57) | 0.322 | 73 (74.49) | 5 (83.33) | 1.000 | 210 (79.85) | 29 (87.88) | 0.270 | 217 (80.07) | 22 (88.00) | 0.486 |
| IABP, n (%) | 10 (10.64) | 0 (0.00) | 0.552 | 10 (10.10) | 0 (0.00) | 1.000 | 10 (3.80) | 0 (0.00) | 0.530 | 10 (3.69) | 0 (0.00) | 1.000 |
| Medical therapy |  |  |  |  |  |  |  |  |  |  |  |  |
| ASA, n (%) | 93 (93.94) | 12 (85.71) | 0.360 | 98 (93.33) | 7 (87.50) | 0.455 | 282 (94.63) | 34 (91.89) | 0.305 | 291 (94.79) | 25 (89.29) | 0.171 |
| Clopidogrel, n (%) | 52 (52.53) | 7 (50.00) | 1.000 | 55 (52.38) | 4 (50.00) | 1.000 | 166 (55.70) | 24 (64.86) | 0.145 | 172 (56.03) | 18 (64.29) | 0.141 |
| Ticagrelor, n (%) | 44 (44.44) | 7 (50.00) | 0.832 | 47 (44.76) | 4 (50.00) | 1.000 | 129 (43.29) | 13 (35.14) | 0.238 | 132 (43.00) | 10 (35.71) | 0.219 |
| ACEI/ARB/ARNI, n (%) | 76 (76.77) | 7 (50.00) | 0.066 | 79 (75.24) | 4 (50.00) | 0.231 | 214 (71.81) | 24 (64.86) | 0.264 | 221 (71.99) | 17 (60.71) | 0.133 |
| BB, n (%) | 86 (86.87) | 13 (92.86) | 1.000 | 91 (86.67) | 8 (100.00) | 0.651 | 262 (87.92) | 31 (83.78) | 0.259 | 269 (87.62) | 24 (85.71) | 0.323 |
| Statin, n (%) | 95 (95.96) | 13 (92.86) | 0.490 | 101 (96.19) | 7 (87.50) | 0.312 | 285 (95.64) | 36 (97.30) | 0.275 | 294 (95.77) | 27 (96.43) | 0.192 |
| Anticoagulant, n (%) | 2 (2.02) | 2 (14.29) | 0.112 | 3 (2.86) | 1 (12.50) | 0.411 | 6 (2.01) | 1 (2.70) | 0.382 | 6 (1.95) | 1 (3.57) | 0.268 |

Abbreviations: DM, diabetes mellitus; HGI, Hemoglobin Glycation Index; BMI, body mass index; CKD, chronic kidney disease; MI, myocardial infarction; PCI, percutaneous coronary intervention; LDL, low-density lipoprotein; HDL, high-density lipoprotein; TG, triglyceride; WBC, white blood cell; hs-CRP, high-sensitivity C-reactive protein; FPG, fasting plasma glucose; HbA1c, haemoglobin A1c; cTnI, cardiac troponin I; LVEF, left ventricular ejection fraction; HR, heart rate; IABP, intra-aortic balloon pump; ASA, acetylsalicylic acid (aspirin); ACEI, angiotensin-converting enzyme inhibitor; ARB, angiotensin receptor blocker; BB, beta-blocker

*p < 0.05, **p < 0.001
